# Frontotemporal White Matter in Adolescents with, and at-Risk for, Bipolar Disorder

**DOI:** 10.3390/jcm3010233

**Published:** 2014-03-10

**Authors:** Sonja M. C. de Zwarte, Jennifer A. Y. Johnston, Elizabeth T. Cox Lippard, Hilary P. Blumberg

**Affiliations:** 1 Department of Psychiatry, Yale School of Medicine, 300 George Street, Suite 901, New Haven, CT 06511, USA; E-Mails: sonja.dezwarte@yale.edu (S.M.C.Z.); jennifer.johnston@yale.edu (J.A.Y.J.); elizabeth.cox@yale.edu (E.T.C.L.); 2 Department of Diagnostic Radiology, Yale School of Medicine, New Haven, CT 06511, USA; 3 Child Study Center, Yale School of Medicine, New Haven, CT 06511, USA

**Keywords:** bipolar disorder, frontal lobe, white matter, development, adolescents, diffusion tensor imaging

## Abstract

Frontotemporal neural systems are highly implicated in the emotional dysregulation characteristic of bipolar disorder (BD). Convergent genetic, postmortem, behavioral and neuroimaging evidence suggests abnormalities in the development of frontotemporal white matter (WM) in the pathophysiology of BD. This review discusses evidence for the involvement of abnormal WM development in BD during adolescence, with a focus on frontotemporal WM. Findings from diffusion tensor imaging (DTI) studies in adults and adolescents are reviewed to explore possible progressive WM abnormalities in the disorder. Intra- and interhemispheric frontotemporal abnormalities were reported in adults with BD. Although evidence in children and adolescents with BD to date has been limited, similar intrahemispheric and interhemispheric findings have also been reported. The findings in youths suggest that these abnormalities may represent a trait marker present early in the course of BD. Functional connectivity studies, demonstrating a relationship between WM abnormalities and frontotemporal dysfunction in BD, and DTI studies of vulnerability in first-degree relatives of individuals with BD, are discussed. Together, findings suggest the involvement of abnormal frontotemporal WM development in the pathophysiology of BD and that these abnormalities may be early trait markers of vulnerability; however, more studies are critically needed.

## 1. Introduction

Bipolar disorder (BD) can be a severe and disabling mood disorder, associated with detrimental outcomes, such as high rates of hospitalizations, substance abuse and suicide [1,2,3]. It is a recurrent illness that is characterized by manic or hypomanic episodes, which may alternate with depressive episodes and periods of normal mood, or euthymia. Dysregulated affective states are central to the acute episodes: euphoric or highly irritable states during mania or sad states during depression. The affective changes are accompanied by shifts in motivation, impulse regulation, energy, and activity levels, in addition to changes in sleep and appetite, implicating the brain regions that subserve emotional regulation and these associated functions.

BD shows a peak in emergence during adolescence and early adulthood [4,5], implicating this epoch as highly important in the development of the disorder. Early onset of BD has been of particular concern because it is associated with a more severe course of the disorder than later presentations, which may have alternate etiologies [6,7]. Therefore, early detection of the illness is of great importance and adolescence has been a major focus within BD-research. Understanding of the development of BD during this epoch could provide insights into the pathophysiology of the disorder. In youth diagnosed with BD, there is a strong overlap in clinical symptoms with other disorders, including attention deficit hyperactivity disorder and major depressive disorder. Misdiagnosis can lead to interventions that have the potential to worsen the outcome of BD [8,9,10]. Therefore, the detection of trait markers early in the disease course might be of importance in adolescents to ensure they receive needed treatments and avoid treatments that could be detrimental.

Convergent evidence supports a central role for altered development of frontotemporal neural systems in BD. Early studies of patients with lesions and seizure foci in anterior cortical and mesial temporal regions, and with lesions in white matter (WM) tracts connecting these regions, reported behavioral symptoms similar to those seen in BD [11,12,13,14,15,16,17,18]. Findings from neuroimaging studies support abnormalities in frontotemporal systems, including abundant findings of abnormal structure and functioning in the amygdala and ventral prefrontal cortex (vPFC) [19]. Recently, studies have suggested that a progression in regional brain abnormalities is present in BD. For gray matter (GM) structures, there is initial evidence that subcortical abnormalities, including the amygdala, may be present by adolescence, while PFC abnormalities, including in the vPFC and more rostral PFC regions, may progress during adolescence and into early adulthood [20,21,22,23,24].

Structural magnetic resonance imaging (MRI) studies and postmortem studies support the involvement of abnormal WM in adults with BD. There is little data available on whether abnormalities in WM appear early and whether they show a progression during adolescence in BD. WM connections continue to develop into adolescence and well into adulthood [25,26,27,28,29,30], suggesting that WM abnormalities in BD might be influenced by developmental changes. In the last decade, the advent of diffusion tensor imaging (DTI) techniques has provided the opportunity to investigate the structural integrity of WM tracts *in vivo* more specifically. Studies have emerged in which this method is applied not only to adults with BD, but also to the study of youths with BD.

This article will review studies that implicate abnormal WM development in frontotemporal neural systems in BD, with a focus on what can be learned about WM in adolescents with BD. DTI findings in adolescents will be discussed in the context of findings in adults to explore the involvement of WM development in the disorder. Although DTI studies will be the focus of the review, studies using other structural imaging techniques will also be included to allow comparisons of DTI findings within the context of other work in the field. Furthermore, functional connectivity studies will be reviewed to explore the relationship between DTI findings and frontotemporal dysfunction in individuals with BD. Finally, this article will examine genetic studies related to WM and neuroimaging studies of first-degree relatives of BD individuals, considered at-risk for the disorder, as BD is a highly heritable illness [31,32] and these studies may help identify possible markers for early detection or even prevention of BD. Together, the studies reviewed support neurodevelopmental mechanisms underlying frontotemporal WM pathology in BD.

## 2. Frontotemporal Neural Circuitry Implicated in BD

Emotional dysregulation is the characteristic feature of BD, suggesting the involvement of abnormalities in frontotemporal brain regions, especially the amygdala and vPFC, which are central to emotional regulation. The amygdala plays an early and important role in emotional processing [33,34,35,36,37], while the vPFC integrates information from the amygdala and other brain regions that provide information about emotional and motivational relevance of stimuli, and synthesizes an adaptive executive feedback to regulate amygdala and other subcortical responses [38,39]. The involvement of frontotemporal abnormalities has been further implicated in BD by behavioral neuroanatomical and neuroimaging studies. Early reports of lesions within the PFC, particularly in the vPFC, provided descriptions of symptoms similar to those seen in BD, including depressive symptoms and manic-like symptoms such as inappropriate euphoria [11,12,13,17,18]. BD-type symptoms were also observed in patients with seizures with foci in the mesial temporal lobe, including in the amygdala [13,15,16]. As the vPFC and the amygdala are highly interconnected brain regions [40], these findings suggest that abnormalities within both the vPFC and amygdala and/or their connections might contribute to BD. Indeed, subsequent findings from structural and functional MRI studies in adults with BD have converged in demonstrating abnormalities in each of these frontotemporal neural system components in individuals with BD [19].

Recent evidence suggests a developmental progression in frontotemporal abnormalities in BD. Subcortical brain structures mature earlier than PFC structures and PFC structures continue to show dynamic maturational changes over adolescence. Therefore, it was theorized that, consistent with the pattern of maturation of brain structures, frontotemporal system abnormalities in BD might emerge earlier in subcortical structures and PFC abnormalities might progress during adolescence [21]. Preliminary structural and functional neuroimaging studies support this progression, with amygdala abnormalities demonstrated in adolescents with BD and PFC abnormalities appearing to progress during adolescence and early adulthood [20,21,22,24]. However, these studies are cross-sectional and must be considered as models of longitudinal outcomes with caution. There is a previous longitudinal study that also supports this model; however, the sample size was small and the significance threshold modest, so findings should be considered preliminary and in need of replication in a larger cohort [23].

In addition to GM developmental changes during adolescence, evidence supports dynamic maturational changes in WM connections between the amygdala and vPFC [27]. Human postmortem and neuroimaging studies have shown that myelination of these connections continues through adolescence and well into adulthood in the normal developing brain [25,26,28,29,30]. The substantial developmental changes in these connections over adolescence and young adulthood suggest that if WM connections are involved in the developmental pathophysiology of BD, they may particularly show changes in their expression during these epochs in the disorder [22,27,29,41,42].

## 3. Frontotemporal WM Connections Implicated in BD

For more than a century, lesions in frontal WM and frontal-subcortical connections have been described in association with both depressive symptoms and manic-like states, including disinhibition and inappropriate excitement and laughter [14]. WM connections within frontotemporal neural systems have since been implicated in the pathophysiology of BD, as the frontotemporal GM structures in which abnormalities have been shown in BD are highly interconnected within these systems. Furthermore, postmortem studies have demonstrated reductions in glia cells, especially oligodendrocytes, and downregulation of genes related to oligodendrocytes and myelination, particularly in frontal brain regions, in individuals with BD [43,44,45,46,47]. These findings suggest abnormalities in myelin synthesis and axonal survival in individuals with BD and implicate the involvement of abnormal WM in the pathology of the disorder.

Frontotemporal WM tracts can be divided into intra- and interhemispheric, *i.e.*, connecting brain structures within one hemisphere or providing connection between the two hemispheres, respectively. Important intrahemispheric WM bundles in the frontotemporal neural circuitry include the uncinate fasciculus (UF) and the cingulum bundle (CB); both carry major connections between the amygdala and vPFC and thus are WM structures especially implicated in BD. Frontotemporal interhemispheric WM abnormalities are also highly implicated in the pathophysiology of the disorder, as the anterior corpus callosum (CC) provides major right-left vPFC connections [48]. Furthermore, studies have identified more widely distributed connections from the amygdala and vPFC, such as to more dorsal PFC, hippocampus, striatum, thalamus, cerebellum and hypothalamus, areas associated with motivational behaviors, biological rhythm and neurovegetative processes [40,49,50,51,52]. Thus, this may explain why impaired amygdala-vPFC connectivity could lead not only to emotional dysregulation, but also to a broader range of symptoms seen in BD.

Studies of structural abnormalities in WM in adults with BD have shown both decreases in volume of WM within ventral frontal regions [22], as well as decreases in CC area and signal intensity, *i.e.*, mean signal intensity thought to reflect myelination [53,54,55]. These implicate both intra- and interhemispheric frontotemporal WM abnormalities in the pathology of BD in adults. Additionally, decreased signal intensity in the CC in BD children and adolescents has been reported [56], suggesting that altered myelination may occur during neurodevelopment in BD and that these WM abnormalities may be an early feature of the disorder.

Other WM abnormalities reported in MRI studies of BD are WM hyperintensities (WMH). These are hyper-intense bright spots that may reflect brain regions of increased water density, possibly because of local altered vascular permeability or other processes [57,58]. However, the etiology of WMH remains unclear. WMH have been associated with aging [58,59], and an increase in their occurrence has also been reported in several studies in adults with BD [60,61,62,63,64,65,66,67,68,69,70]. These findings might suggest that the accumulation of WMH might be associated with aging processes in the disorder [71]. Furthermore, an association between increased WMH and previous suicide attempts has been reported [62], suggesting the involvement of WMH in risk for suicide attempts in BD individuals. Some of these studies reported WMH in the deep frontal WM, further suggesting that tracts connecting fronto-cortical and subcortical regions are affected in the disorder [61,68]. Evidence related to WMH in youths with BD is contradictory. In some studies of children and adolescents with BD, significant increases in WMH number were not detected [72,73]. There have also been reports of increases in WMH in children and adolescents with BD [74,75]. This suggests that WMH may involve pathophysiological processes other than aging or may be associated with particular subtypes of BD, such as early onset. However, the involvement of WMH early in the disorder remains unclear.

## 4. DTI Studies Implicating Frontotemporal WM Abnormalities in Adolescents with BD

DTI studies have started to focus on structural integrity differences between WM in adolescents with BD, compared to healthy comparison (HC) adolescents (Table 1). DTI studies provide noninvasive measures of the organization of WM [76]. Fractional anisotropy (FA) is a common DTI measure that provides information on the structural integrity and coherence of fibers within WM regions [77]. Some studies report additional measures to provide more insight on diffusivity in WM, such as apparent diffusion coefficient (ADC) or mean diffusivity (MD), radial diffusivity (RD) and axial diffusivity (AD); however, FA is the most consistently reported measure and therefore the main focus of this review.

Frontotemporal WM abnormalities implicated in BD, both intra- and interhemispheric, have been reported in many adult DTI studies. Reduced UF and neighboring orbitofrontal WM integrity [78,79,80,81,82,83,84,85,86,87,88] and reduced anterior CB integrity [86,89,90,91,92] have been among the most consistent WM integrity findings in adults with BD. Studies of adults with BD have also shown intrahemispheric WM abnormalities in connections between dorsal frontal regions, to regions such as the striatum or thalamus via connections within structures, including the anterior limb of the internal capsule (ALIC) [82,87,93,94]. More abundant interhemispheric findings include reduced structural integrity in the anterior CC in adults with BD, and supports abnormal interhemispheric frontotemporal circuitry in the disorder [70,87,88,94,95,96,97,98,99]. In addition to tensor-based diffusion MRI studies, recent research in adults with BD has also utilized alternative methodologies, such as high-angular resolution diffusion imaging (HARDI) and diffusion spectrum imaging (DSI), which are able to further parse heterogeneous diffusion directions and crossing fiber tracts [99,100]. These techniques are promising and may potentially provide additional insight into WM abnormalities in BD.

**Table 1:** Diffusion tensor imaging (DTI) studies of children and adolescents with bipolar disorder and/or at-risk for bipolar disorder.

Authors and Year	Subjects	Mean Age	Age Range (years)	DTI Measures	Analysis Type	Regions of Interest	Findings and Overall Significance Levels	
		(year ± SD)						
Studies of children and adolescents at-risk for BD								
Versace *et al*. 2010 [101]	20 AR-BD 25 HC	13.2 ± 2.5 13.9 ± 2.6	8–17	FA, RD, L1	TBSS	Whole brain	AR-BD > HC: FA in left CC body, L1 in right ILF	
							AR-BD < HC: RD in left CC body, right ILF	
							Significant group-by-age interaction:	
							HC: FA increases/RD decreases with age in left CC body; RD decreases with age in right ILF; L1 increases with age in right ILF	
							AR-BD: FA decreases/RD increases with age in left CC body; RD no relation with age in right ILF; L1 decreases with age in right ILF	
							AlphaSim corrected *p* < 0.05	
Studies of children and adolescents both with BD and at-risk for BD								
Frazier *et al*. 2007 [102]	7 AR-BD 10 BD 8 HC	8.9 ± 3.0 9.2 ± 3.0 9.2 ± 2.4	4–12	FA	Voxel-based	*A priori* CPC and SLF, and whole brain	BD < HC: FA in bilateral CPC and SLF, right CC body, left OF WM	
							AR-BD < HC: FA in bilateral SLF	
							BD < AR-BD: FA in bilateral CPC	
							*p* < 0.05 Bonferroni corrected	
Studies of children and adolescents with BD								
Adler *et al*. 2006 [103]	11 BD 17 HC	14 ± 2	10–18	FA, trace ADC	ROI	Frontal and posterior regions	BD < HC: FA in bilateral superior frontal WM tracts	
							*p* < 0.01 uncorrected	
Kafantaris *et al.* 2009 [104]	26 BD 26 HC	16.0 ± 1.5 15.3 ± 1.5		FA, ADC	Voxel-based	Whole brain	BD < HC: FA in right OF WM, bilateral temporal lobes, left occipital lobe	
							BD > HC: ADC in bilateral subgenual region, bilateral precuneus, left postcentral gyrus, left temporal and right occipital lobes	
							*p* < 0.005 uncorrected, cluster size ≥100	
Pavuluri *et al*. 2009 [105]	13 BD 13 ADHD 15 HC	14.8 ± 2.5 13.4 ± 3.0 13.7 ± 2.7		FA, ADC, r-FCI	ROI and voxel-based	ACR, ALIC, CB, CC (splenium), ILF, PLIC, SLF, SRI	BD and ADHD < HC: FA in ACR, r-FCI in CC splenium	
							BD and ADHD > HC: ADC in CC splenium	
							*p* < 0.05 uncorrected	
Barnea-Goraly *et al*. 2009 [106]	21 BD 18 HC	16.1 ± 2.7 14.5 ± 2.7	9–18	FA, trace values (average diffusivity), ADC	TBSS	Whole brain	BD < HC: FA in CC, PCR, left mid-posterior CB, fornix, fibers from fornix to thalamus	
							*p* < 0.05 corrected	
Gönenç *et al*. 2010 [107]	10 BD (8 in study) 10 HC (8 in study)	3M: 16.6 ± 4.87 F: 11.8 ± 4.5 6M: 10.6 ± 4.0 4F: 14.3 ± 1.9	6–18	FA, trace diffusivity, RD, AD	ROI	Bilateral CPC	BD < HC: FA left CPC	
							BD > HC: left and right trace and left RD in CPC	
							*p* < 0.05 uncorrected	
Saxena *et al*. 2012 [108]	10 BD 10 HC	13.9 ± 3.6 13.6 ± 3.6	7–17	FA	TBSS	5 CC	BD < HC: FA genu CC and AC	
						subdivisions, AC	*p* < 0.001 uncorrected, *p* < 0.05 small-volume FDR correction	
Gao *et al*. 2013 [109]	18 BD 18 HC	15.1 ± 1.8 14.1 ± 1.6	10–18	FA	TBSS	Whole brain	BD < HC: FA right anterior CB	
							*p* < 0.05 FDR correction	
**Abbreviations**								
							PCR	Posterior corona radiate
AC	Anterior commissure			CPC	Cingulate-paracingulate		PLIC	Posterior limb of the internal capsule
ACR	Anterior corona radiate			DTI	Diffusion tensor imaging		OF	Orbitofrontal
AD	Axial diffusivity			F	Female		r-FCI	Regional fiber coherence index
ADC	Apparent diffusion coefficient			FA	Fractional anisotropy		RD	Radial diffusivity
ADHD	Attention deficit hyperactivity disorder			FDR	False discovery rate		ROI	Region of interest
ALIC	Anterior limb of the internal capsule			HC	Healthy comparison		SD	Standard deviation
AR-BD	At-risk for BD, *i.e.*, having a first-degree relative with BD			ILF	Inferior longitudinal fasciculus		SLF	Superior longitudinal fasciculus
BD	Bipolar disorder			L1	Longitudinal diffusivity		SRI	Superior region of the internal capsule
CB	Cingulum bundle region			M	Male		TBSS	Tract-based spatial statistics
CC	Corpus callosum						WM	White matter

Studies of children and adolescents with BD have also shown findings of reduced intrahemispheric structural integrity in ventral frontal WM regions, compared to HC children and adolescents [102,104] (Table 1). Decreased FA values have been reported in pericingulate regions, including the CB, in children and adolescents with BD [102,107,109]. These decreases in WM structural integrity include findings in prepubertal children [102,107]. This might be an indication that intrahemispheric frontotemporal WM abnormalities are already present in childhood, representing some of the earliest markers of the disorder.

Abnormalities in additional tracts have also been demonstrated in children and adolescents with BD, including tracts to other connection sites of frontotemporal circuitry, such as to more dorsal frontal regions, basal ganglia, thalamus and posterior association cortices [102,105,106]. Reduced structural integrity in superior frontal regions has been reported in BD adolescents [103]. Reductions of FA values have also been reported in frontal projections through the anterior corona radiata in children and adolescents [105,106], and in a group that included both adolescents and young adults [98], with BD. In contrast to findings in adults, abnormalities in the ALIC have not been a consistent finding in children and adolescents with BD. Studies have reported both no differences [105] and reduced structural integrity of the ALIC in children and adolescents with BD compared to HC youths [110]. The latter study included both adults and youths with BD and compared early- and late-onset of BD and reported lower FA values in the early-onset group, suggesting that early- and late-onset BD might represent different subtypes within the bipolar spectrum with different pathophysiologies, potentially accounting for some conflicting results in the literature.

Findings of frontotemporal interhemispheric WM abnormalities in adolescents with BD have also started to emerge. Lower FA values of interhemispheric connections in a group with both children and adolescents with BD have been found in the anterior commissure (AC) [108]. Decreases in structural integrity in the anterior CC have been reported in prepubertal children and adolescents [106,108]. The AC and the anterior CC link the right and left temporal lobes and prefrontal lobes respectively, and therefore could play a role in the frontotemporal neural circuitry suggested in BD. Findings suggest that reduced integrity of frontotemporal interhemispheric WM bundles might be present early in the disease course.

## 5. Functional Connectivity Studies and the Relationship between DTI Results and Dysfunction in BD

Functional connectivity neuroimaging studies further implicate the involvement of abnormal frontotemporal WM connections in BD. Functional connectivity MRI measures can be derived from the degree that activity is coordinated in time between different brain regions. Functional neuroimaging studies have provided support for trait abnormalities in functional connectivity between the amygdala and vPFC regions in both adults [81,111,112,113,114,115,116] and children and adolescents [117,118,119] with BD across mood states.

Several studies have aimed to examine the relationship between DTI results and dysfunction in BD. An investigation combining DTI and functional connectivity data showed an association between reduced structural integrity in the UF with decreases in the functional connectivity between the vPFC and the amygdala during processing of emotional stimuli by adults with BD [81]. This multimodal neuroimaging study suggests that the WM aberrations may contribute to disruptions in the ability of the frontotemporal brain structures to work together in the regulation of responses to emotional stimuli. 

Hemispheric lateralization of processing of positive and negative emotions is evident in normal affective processing, and the balance between the hemispheres has been proposed to be important in healthy emotional regulation [120]. A low frequency resting state functional connectivity study showed increased interhemispheric correlations between left and right vPFC in adults with BD, relative to healthy individuals [111]. This finding suggests that interhemispheric abnormalities might contribute to impaired hemispheric integration and the hemispherically-lateralized dysfunction in acute mood states of the disorder [48,111].

A functional connectivity study investigating children and adolescents with BD showed less functional connectivity between the amygdala and posterior association cortices during an emotional face identification task [121]. This finding suggests that youth with BD may have impaired communication in neural systems critical to processing faces and emotional stimuli and that WM connections to posterior regions may be early abnormalities in BD. As connections from posterior associations structures to amygdala may carry information used to associate stimuli with emotional information [122], abnormalities in these connections could contribute to abnormalities in the development of emotional associations to environmental stimuli. More multimodal studies are needed to further delineate the relationship between WM abnormalities and dysfunction in BD.

## 6. Association of WM Abnormalities with Genetic Risk for BD

Increasing evidence that genes related to WM and frontotemporal connectivity are associated with BD further implicate frontotemporal neural circuitry in the disorder. BD has strong genetic contributions with heritability estimates varying from 40% to 70% [123]. Studies have suggested that genes involved in cytoarchitecture of frontotemporal WM structures might be associated with an increased risk of BD [124]. For example, the neuregulin 1 (NRG1) gene plays a key role in neurodevelopmental processes in WM brain connections, such as in axonal guidance and myelination [125,126,127]. NRG1 is suggested to influence the susceptibility to BD and seems especially associated with psychotic features [128]. Abnormal frontotemporal WM volume, including in the CB and regions in the CC, has been found to be associated with a single nucleotide polymorphism (SNP) in the NRG1 gene (rs35753505) in adults with BD [129]. DTI evidence showed that another NRG1 SNP (rs6994992) is associated with reduced WM density and integrity in the ALIC [130], suggesting that NRG1 may increase susceptibility to psychopathology by altering connections between PFC and other brain regions. Another gene of particular interest in BD is the CACNA1C gene, which has been reported in genome-wide association studies to be related to BD [131,132]. The CACNA1C gene is implicated in the development and plasticity of the frontotemporal neural circuitry [133]. CACNAIC rs1006737 SNP variation has been associated with altered frontotemporal functional connectivity between the amygdala and vPFC [134]. This finding implicates variations in gene expression in the neural circuitry associated with BD, and that altered gene expression associated with BD may lead to abnormalities in WM connections, including in their development and plasticity, as well as in associated frontotemporal functional connectivity.

The high heritability rates in BD are supported by several longitudinal studies, showing offspring of individuals with BD to be at greater risk to develop BD [135,136]. First-degree relatives of affected BD individuals are considered to have more than a ten-fold higher risk of developing BD than the general population [137]. Thus, youths at-risk for BD (AR-BD), as they have a first-degree relative with BD but they themselves have not yet developed BD, are of particular interest to investigate. The presence of neurodevelopmental abnormalities in AR-BD youth, prior to the onset of acute episodes of the disorder, was suggested by a prospective study of a large cohort [138]. AR-BD youths might show brain differences that are apparent even before onset of mood episodes, revealing abnormalities associated with vulnerability to the disorder and minimizing confounds, such as the possibility that abnormalities are a result of having experienced an acute episode or medication exposure.

Structural MRI studies in AR-BD adults have shown reduced WM volume in the left hemisphere [139] and WMH abnormalities [140,141], suggesting that WM disconnectivity might also be present in AR-BD individuals. However, only limited DTI studies have to date explored structural integrity in AR-BD individuals with BD.

In DTI studies, frontotemporal structural integrity abnormalities have been reported in both adults with BD and their AR-BD adult relatives, both showing decreased FA values in the right UF [87]. Reductions of structural integrity in the ALIC and in frontal connections to posterior association cortices have also been reported in adult AR-BD individuals [87,94,142,143]. Decreased structural integrity found in the CC in BD individuals has not been detected in their relatives [87]; however, increased genetic liability for BD was reported to be associated with a trend towards reduced FA in the anterior CC, with intermediate values for AR-BD adults [94]. This suggests the possibility that intrahemispheric frontotemporal connections are more associated with genetic heritability than interhemispheric WM connections. The frontal system WM findings in AR-BD are consistent with reports of cognitive dysfunction in AR-BD adults [144]. Future research to combine tests of cognitive and behavioral functions with neuroimaging may help to identify relationships between cognitive dysfunction and underlying WM abnormalities.

To date, only limited numbers of studies have been reported that explore WM in AR-BD youths (Table 1). One study reported altered WM in the left CC during early adolescence in AR-BD youths, compared to youths with no family history of BD, with the AR-BD youths showing decreases in FA with age, while the low-risk youths showed an increase of FA with age [101], suggesting altered WM developmental trajectories in the AR-BD adolescents. Another study reported decreased structural integrity in bundles connecting frontal cortices with posterior association cortices in both prepubertal children with BD and AR-BD prepubertal children [102]. These results raise the intriguing possibility that abnormalities in WM connections may provide an early marker for vulnerability to BD. However, due to the limited DTI research conducted in AR-BD subjects, it remains unclear whether WM abnormalities are involved in the vulnerability for developing BD and whether they could potentially be a biomarker for the disorder.

## 7. Conclusions

While GM volumetric and functional changes have been a focus of neuroimaging research in BD, recent neuroimaging data have been converging to suggest WM abnormalities may be important in the developmental pathophysiology of BD and have potential as early biomarkers. Intrahemispheric WM abnormalities in UF and CB are especially implicated in the disorder, and additional abnormalities in connections to dorsal frontal regions, striatum and thalamus, as well as to posterior association cortices, have also been shown in adults and adolescents. Interhemispheric anterior CC abnormalities have also been repeatedly shown in adults with the disorder, although less evidence is available in adolescents. WM abnormalities are associated with frontotemporal system dysfunction, as well as associated behaviors. Genetic variations associated with both the development of WM connections and with BD susceptibility have also shown association with frontotemporal structural and functional connection abnormalities, suggesting potential genetic mechanisms that may underlie connection abnormalities. The intrahemispheric abnormalities show promise as possible early markers of vulnerability to BD. There is less data to support interhemispheric abnormalities in vulnerability.

More research is critically needed. Longitudinal studies of adolescents with BD could help to identify the processes involved in the neurodevelopment of BD, contributing to understanding of the progression of abnormalities and the factors that contribute to them. Longitudinal studies of AR-BD children are especially needed. These could help to identify neurodevelopmental processes involved in the transition from risk to developing the disorder for children who go onto develop BD, as well as protective processes for those who do not. Increased sample sizes, as well as consideration of various heterogeneous demographic and clinic features, are needed. It will be important to investigate associations between specific regional findings with symptom and behavioral domains to provide a dimensional understanding of neuropathological mechanisms of BD.

Measures assessed in imaging studies have been limited, such as a focus primarily on FA in DTI studies. As additional measures may help to clarify the types of pathophysiological processes involved have been informative in studies of adults, and have shown some differences in studies of youths, they will be important to include in future studies. Previous studies have varied in other aspect of the imaging acquisition and analyses methods, including variation in the statistical thresholds used. The field will benefit from increasingly sensitive and specific measures of white matter features, and of studies with larger samples with more stringent statistical thresholds.

In summary, research on WM in BD supports an important role for frontotemporal WM. Neurodevelopmental abnormalities that affect trajectories of WM development during adolescence are implicated in the emergence of BD during this epoch. Future studies might reveal important insights into the pathophysiology of BD and identify brain differences and mechanisms to target for early identification, intervention and prevention strategies.
